# The Role of the Complement C3‐Hippocampus Pathway in Relation With Mood Symptoms in Offspring of Parents With Bipolar Disorder

**DOI:** 10.1111/bdi.70056

**Published:** 2025-09-01

**Authors:** Shiyun Wu, Zhongwan Liu, Robin Shao, Wenjin Zou, Xiaoyue Li, Weicong Lu, Jinyong Chen, Suk‐Yu Yau, Kangguang Lin

**Affiliations:** ^1^ Department of Affective Disorder The Affiliated Brain Hospital of Guangzhou Medical University Guangzhou People's Republic of China; ^2^ Key Laboratory of Neurogenetics and Channelopathies of Guangdong Province and the Ministry of Education of China Guangzhou Medical University Guangzhou People's Republic of China; ^3^ Department of Psychology The University of Hong Kong Hong Kong People's Republic of China; ^4^ Lecong Hospital of Shunde Foshan People's Republic of China; ^5^ Department of Rehabilitation Sciences, Faculty of Health and Social Sciences The Hong Kong Polytechnic University Hong Kong People's Republic of China; ^6^ School of Health and Life Sciences University of Health and Rehabilitation Sciences Qingdao People's Republic of China

**Keywords:** bipolar disorder, C3, hippocampus, offspring, subthreshold symptoms

## Abstract

**Objective:**

Accumulative research indicates key roles of the peripheral inflammation system and hippocampal function in major mood disorders. The complement system modulates inflammatory function and is abnormal in mood disorders, but its precise neural pathway remains unclear. This study investigates the interrelations among complement component 3 (C3) levels, hippocampal function, and mood symptoms among offspring of bipolar disorder (BD) parents who carry familial risk of mood disorders.

**Method:**

We recruited unaffected BD offspring with (symptomatic offspring, SO, *N* = 31) or without (asymptomatic offspring, AO, *N* = 39) subthreshold symptoms, and matched healthy controls (HC, *N* = 41). Peripheral C3 levels were measured, and resting‐state fMRI was conducted to assess hippocampal functional connectivity (FC). Spectral dynamic causal modeling (spDCM) was conducted to verify the directionality of the hippocampal FC.

**Results:**

The SO group exhibited significantly lower peripheral C3 levels (*F*_2,108_ = 23.651, *p* < 0.001) and reduced left hippocampus‐left cerebellum FC (*F*_2,108_ = 8.541, *p* < 0.001) compared to both the AO and HC groups. Furthermore, the left hippocampus‐left cerebellum FC partially mediated the relationship between C3 levels and depressive symptoms in the SO group (bootstrapping 95% CI = −4.1168 to −0.1569), but not in AO (bootstrapping 95% CI = −0.3479 to 0.1317) or HC (bootstrapping 95% CI = −0.3297 to 0.0885). The left hippocampus‐left cerebellum FC was bidirectional in all 3 groups.

**Conclusion:**

Our findings indicate a C3‐hippocampus‐depressive symptom pathway might underpin the particular high vulnerability of individuals with both familial and symptomatic risks of mood disorders. This evidence provides new neuroinflammatory markers and targets for early identification and intervention of these individuals.

## 1 Introduction

Bipolar disorder (BD) is a severe psychiatric illness characterized by recurrent episodes of depression and hypo/mania, affecting approximately 2.4% of the global population [1]. The risk of developing BD is notably higher among offspring of patients, increasing by 5–10 times compared to the general population [2]. A proportion of BD offspring exhibit early subthreshold symptoms such as depression and hypo/mania, which often precede the onset of full‐blown BD and represent a particularly high‐risk population [3]. However, other BD offspring can remain asymptomatic and free of clinical diagnosis; thus, they are considered to possess resilience against the familial risk [4]. Understanding the biological mechanisms underlying the risk and resilience processes of BD offspring offers value to early identification and prevention of the disease.

Recent studies underscore the pivotal role of neuroinflammation and immune dysfunctions in the etiology of BD [5]. Previous research showed that symptomatic BD offspring exhibit higher levels of peripheral inflammatory markers, including C‐reactive protein (CRP) and interleukin‐6 (IL‐6) compared to asymptomatic offspring (AO) [6, 7]. However, the underlying process for such an increase in inflammatory processes remained unclear. It is known that the body's complement system modulates peripheral inflammatory responses and the release of IL‐6 and CRP [8, 9]. Furthermore, as the core molecule of complement proteins, complement component 3 (C3) is the intersection of the complement activation pathway and plays a pivotal role in synaptic pruning and brain development [10]. C3 convertase cleaves C3 into C3a and C3b, of which C3a is an allergic toxin that can stimulate chemotaxis and leukocyte activation to regulate pro‐inflammatory responses and lead to the release of factors such as IL‐6 [11]. Past studies reported that the expression of C3‐related genes has a significant protective effect against schizophrenia [12] and have identified that lower C3 levels are correlated with an increased risk of schizophrenia [13]. Furthermore, reduced C3 levels can exacerbate psychotic symptoms as well as depression and anxiety [14, 15]. Notably, recent evidence suggests changes in C3 precede the onset of psychiatric disorders [16], indicating the potential utility of C3 as a biomarker for BD risk. However, the specific role of C3 in high‐risk BD offspring remains to be elucidated.

The body's complement system can have a profound influence on brain function. Existing evidence indicates that C3 is implicated in hippocampal synaptic pruning and remodeling, potentially contributing to hippocampal structural and functional deficits [17]. The hippocampus receives and sends neural inputs from other important brain structures involved in emotion processing and regulation functions, such as the ventral prefrontal cortex, limbic regions, and cerebellum [18, 19]. Numerous studies have further shown that abnormalities in hippocampal function are considered a potential BD endophenotype feature [4, 20]. A genome‐wide association (GWAS) study found that BD risk alleles were enriched in the hippocampus [21]. Furthermore, our previous research showed that hippocampal resting‐state functional connectivity (rsFC) has high predictive utility for the onset of BD and future mood disorders among offspring of BD parents [20]. Other studies also showed abnormalities of hippocampal rsFC with brain structures such as the cerebellum [4] and posterior cingulate cortex [20] in association with BD familial risk.

However, there is a paucity of evidence on the importance of a C3‐hippocampus pathway in underlying BD familial and symptomatic risks. Such a pathway is supported by previous research indicating the profound modulatory influence of C3 on the hippocampus [22], and an abnormality in the C3‐hippocampus system may underpin the brain network dysfunctions observed in major psychiatric disorders such as BD. Although C3 has demonstrated a role in regulating emotion‐related behaviors [23], it remained to be elucidated whether the particularly high‐risk BD offspring with subthreshold symptoms would exhibit an abnormality in the interaction of C3 and hippocampal function.

In this study, we primarily examined the interrelations between C3 level, hippocampal function, and mood symptoms among BD offspring, and tested whether the C3‐hippocampus pathway was altered in particularly high‐risk offspring with both familial and symptomatic risks. Based on existing evidence, we hypothesized that symptomatic BD offspring would show both lower C3 level and abnormal hippocampal functional connectivity than asymptomatic BD offspring and HC. Critically, the pathway in which C3 modulates mood symptoms via influencing the hippocampal function would be altered in symptomatic offspring (SO) compared to asymptomatic BD offspring and HC.

## 2 Materials and Methods

### 2.1 Participants

The present study was part of the Recognition and Early Intervention on Prodromal BP (REI‐PBD) project (ClinicalTrials.gov Identifier: NCT01863628). All participants were recruited from the hospital and community through advertisements. We recruited BD offspring with no BD diagnosis who manifested subthreshold symptoms (SO, *N* = 37), BD offspring without subthreshold symptoms (AO, *N* = 47), and matched healthy controls (HC, *N* = 41). This study was approved by the Institutional Review Board of the Affiliated Brain Hospital of Guangzhou Medical University. All participants and/or their guardians provided written informed consent.

The BD offspring groups had at least one biological parent diagnosed with bipolar type I/II according to the DSM‐IV criteria. For the SO group, the subthreshold symptoms were defined as at least 1 of the following: (1) two or three hypomanic symptoms (e.g., irritable mood, grandiosity, and increased talkativeness) lasting for at least 4 days, while not meeting the DSM‐IV hypomania episode criteria; (2) two to four depressive symptoms (e.g., depressed mood, loss of pleasure or interest, insomnia) lasting for at least 1 week, while not meeting the DSM‐IV major depressive disorder (MDE) criteria; (3) one or more attenuated psychotic symptoms present for at least 10 min for each manifestation, occurring 2–7 times per week for at least 3 months. The attenuated psychotic symptoms included: odd ideas and beliefs, ideas of reference, bizarre thoughts or speech, unusual perceptual experiences, grandiosity, suspicious ideas, paranoid ideas, odd mannerisms, hallucinations, disorganized/catatonic behaviors; (4) two or more hyperactivity/impulsivity symptoms of attention deficit hyperactivity disorder (ADHD) as observable by teachers, peers, and/or parents. All SO participants who manifested ADHD or attenuated psychotic symptoms also needed to display either subthreshold depressive or hypomanic symptoms as outlined above. Individuals with any DSM‐defined psychiatric disorders were excluded, and no participant was on psychotropic medication. No participant in the AO or the HC group exhibited subthreshold symptoms as outlined above. The HC group had no first‐ or second‐degree family history of psychiatric disorders. No participant showed mental retardation, severe medical illness, intake of psychoactive drugs or thyroxine, regular use of substances or alcohol, or MRI incompatibility.

### 2.2 Clinical Assessment

We administered the Kiddie Schedule for Affective Disorders and Schizophrenia‐Present and Lifetime Version (K‐SADS‐PL) on participants under 18 years of age [24] or the Structured Clinical Interview for DSM‐IV Axis I Disorders (SCID‐I/P) on adult participants for screening psychiatric disorders, respectively. We then administered the 74‐item symptom checklist modified from the Bipolar Prodrome Symptom Scale‐Retrospective: Patient Version (BPSS‐RPt) to measure participants' current and past symptoms. Participants' depression and mania symptom levels were assessed by the Hamilton Depression Rating Scale (HAMD) [25] and the Young Mania Rating Scale (YMRS) [26]. Detailed information about participant assessment is included in the Data S1.

### 2.3 Peripheral C3 Level Measurement

All participants' serum C3 levels were isolated from whole blood samples by centrifugation at 3000 *g* for 10 min at 4°C, and were stored in 1 mL aliquots at −80°C. Levels of C3 were measured by the Bio‐Plex cytokine assay according to the manufacturer's instructions. All samples were assayed in triplicate.

### 2.4 MRI Protocol Procedure and Preprocessing

All participants' imaging data were acquired on a Philips Achieva X‐series 3.0 Tesla scanner with an 8‐channel SENSE head coil. Resting state images were acquired axially with a gradient echo‐planar imaging sequence. The parameters were: TR = 2000 ms, TE = 30 ms, FOV = 220 × 220 mm^2^, voxel sizes = 3.44 × 3.44 × 4 mm^3^, matrix size = 64 × 64, slice number = 33, slice thickness = 4 mm, slice gap = 0.6 mm, 240 volumes. T1‐weighted images were acquired with a three‐dimensional sagittal fast field echo sequence, with the following parameters: TR = 8.2 ms; TE = 3.7 ms; FOV = 256 × 256 mm^2^; voxel sizes = 1 × 1 × 1 mm^3^; matrix size = 256 × 256; slice number = 188.

Resting‐state fMRI data preprocessing was conducted with the MATLAB 2019b software using the DPARSF V7.0 toolbox (Data Processing & Analysis of Brain Imaging V7.0, http://rfmri.org/DPARSF). Firstly, we removed the initial five volumes, and the residual 235 volumes were corrected for slice‐timing and head motion. The white matter signal, cerebrospinal fluid signal, and the Frison‐24 motion parameters were then regressed out from the signals as nuisance variables. The functional images were then resampled to 3 × 3 × 3 mm^3^ resolution, normalized to the Montreal Neurological Institute (MNI) space using the Diffeomorphic Anatomical Registration Through Exponentiated Lie algebra (DARTEL), smoothed by a 6 mm full‐width‐at‐half‐maximum (FWHM) Gaussian kernel, and temporally band‐pass filtered at 0.01 to 0.08 Hz. The choice of 6‐mm FWHM in spatial smoothing is one of the most common approaches in fMRI data analysis and was recommended by previous research [27]. Smoothing using 6‐mm FWHM was previously found to improve signal‐to‐noise ratio in the imaging data and signal detection power [28] and was adopted in empirical validation studies that compared among data processing pipelines [29, 30]. Recent study on BD patients also adopted 6‐mm FWHM smoothing for resting‐state signals [31]. Furthermore, it should be noted that the TFCE procedure we used to compute result significance is considered to show little reliance on the extent of spatial smoothing [32]. Fourteen participants were excluded due to excessive head motion, resulting in 41 HC, 31 SO, and 39 AO in the data analysis (Data S1).

### 2.5 Seed‐Based FC Analysis

We first selected the left and the right hippocampus as two regions of interest (ROIs) using the WFU‐PickAtlas toolbox based on the Automated Anatomical Labeling template (AAL). Then, we extracted the averaged timeseries of each ROI, and the FC maps were obtained by calculating the Pearson correlations between the timeseries of the ROI and the timeseries of each voxel outside the ROI. Fisher's *r*‐to‐*z* transformation was conducted to convert correlation maps into *z* maps.

We then compare the hippocampus FC maps among AO, SO, and HC groups using one‐way ANOVA implemented in Statistical Parametric Mapping toolbox (SPM12, Wellcome Department of Cognitive Neurology, London, UK). Since we were primarily interested in the unique difference between the SO on one hand and the AO and HC on the other hand, we conducted conjunction analysis based on the SO vs. HC and the SO vs. AO contrasts. Specifically, following previously published guidelines [33], we first computed the results of SO vs. HC. We then drew an 8‐mm sphere centered on the peak coordinate of the resultant cluster and evaluated the SO vs. AO contrast within this sphere. Statistical thresholds were set as Family‐wise‐error (FWE)‐corrected *p* < 0.05 using the nonparametric threshold‐free cluster enhancement (TFCE) [32] method with 5000 permutations. Lastly, we extracted the FC values of the significant voxels for follow‐up analyses, as detailed below.

### 2.6 Spectral DCM Analysis

We performed spectral dynamic causal modeling (spDCM) analysis to investigate the directionality of the FC causal interactions. The analysis followed the pipeline as described below: (1) defining the seed regions as (i) the left hippocampus; (ii) a 6‐mm sphere centered at the peak coordinate of the regions where FC with the left hippocampus showed significant between‐group effects; (2) extracting the first eigenvariate of the timeseries of the seed regions, correcting for mean, head motion, white matter, and cerebrospinal fluid signals; (3) specifying three DCM models: hippocampus‐to‐target, target‐to‐hippocampus, and bidirectional; (4) performing family‐level Bayesian model selection [34] to obtain the winning model in each of the three groups; (5) estimating the amplitudes of the connections using Bayesian model average, which weights each model based on its evidence; and (6) conducting general linear models to test whether the connectivity strengths showed significant group effect after controlling for individuals' age and sex.

### 2.7 Statistical Analysis

#### 2.7.1 Demographic, Clinical Variables and C3 Level Analysis

We performed statistical analyses using SPSS version 26.0 (Chicago, IL) to compare demographic and clinical variables between SO, AO, and HC groups. Data normality and variance were respectively checked using the Kolmogorov–Smirnov test and Levene's test. For continuous variables, we utilized one‐way ANOVA if the data satisfied normality and showed no heteroscedasticity; otherwise, the nonparametric Kruskal–Wallis test was conducted. For binary variables, we utilized the chi‐square test. Participants' C3 levels were ln‐transformed and analyzed using the Kruskal–Wallis test while controlling for age and sex. Post hoc pairwise comparisons with Bonferroni correction were conducted in case of significant ANOVA results. The statistical threshold was *p* < 0.05, two‐tailed.

#### 2.7.2 C3‐Brain‐Clinical Analysis

We assessed the relationships between C3 level, hippocampus FC, and clinical symptoms in the total participant sample. In cases of data non‐normality, we conducted Spearman's rank‐based correlation analysis while controlling for age and sex. The correlation analysis results were further corrected for multiple testing using the Holm‐Bonferroni method.

We then assessed whether any relationship between C3 level and clinical symptoms was mediated by hippocampus FC, and whether this mediation effect varied across groups. Mediation analysis was conducted using the PROCESS macro (v.4.1) in SPSS, which utilizes a bootstrapping method [35]. Following that, a moderated mediation analysis was conducted to assess whether the group variable significantly moderated the mediation effect. All analyses controlled for age and sex as nuisance variables. We used a bias‐corrected procedure to bootstrap 5000 samples, which produced 95% confidence intervals (CIs). Significance was determined on whether the CI excluded zero. While there is no consensus on how many times the bootstrapping procedure should be performed, the user manual for the PROCESS macro recommends using 5000 samples [36]. Extensive past evidence indicates that bootstrapping 5000 times is a reasonably conservative approach that improves CI estimation with minimal extra burden for computational cost [37, 38]. Recent research which investigated mediating relationships in BD also employed 5000‐sample bootstrapping [39].

## 3 Results

### 3.1 Demographic, Clinical Variables and C3 Analysis

**FIGURE 1:**
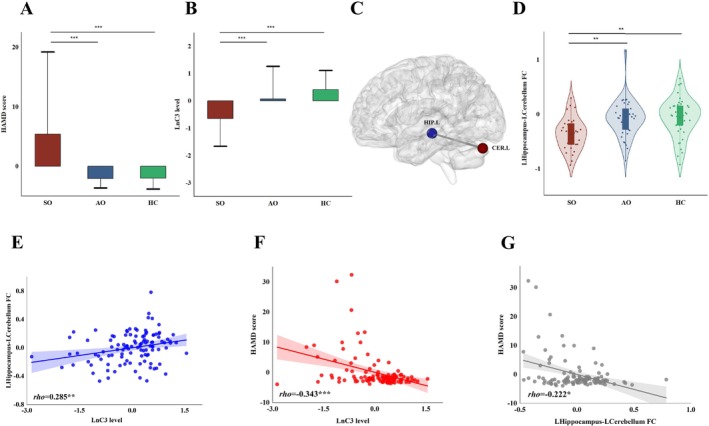
Between‐group differences and interrelations between C3, hippocampus‐cerebellum FC, and HAMD scores. Compared to both AO and HC groups, the SO group showed a significantly higher HAMD score (A, both *p*_corrected_ < 0.001) and a lower C3 level (B, both *p*_corrected_ < 0.001), while no difference was observed between the AO and HC groups (*p*_corrected_ > 0.05). (C) Conjunction analysis revealed a common left cerebellum region that showed reduced FC with the left hippocampus seed in the SO group compared to both the HC and the AO groups (*p*_FWE_ < 0.05, D). In the total participant sample, the C3 level positively correlated with the left hippocampus‐left cerebellum FC (E, Spearman *rho* = 0.285, *p*_corrected_ < 0.005) and negatively correlated with the HAMD score (F, Spearman *rho* = −0.343, *p*_corrected_ < 0.001), whereas participants' left hippocampus‐left cerebellum FC correlated negatively with the HAMD score (G, Spearman *rho* = −0.222, *p*_corrected_ < 0.05). Nuisance variables including age and sex were controlled for in all analyses. AO, asymptomatic offspring; CER.L, Left Cerebellum; HC, healthy controls; HIP.L, left hippocampus; SO, symptomatic offspring. ***p_corrected_ < 0.001; **p_corrected_ < 0.005; *p_corrected_ < 0.05. Please refer to Figure S1 for scatter plots including group‐specific correlation and 95% CI.

The current study involved 111 participants (mean age = 17.74 ± 4.36 years), comprised of 31 SO, 39 AO, and 41 HC. The three groups were matched on age, sex, and years of education (all *ps* > 0.05, Table 1). The SO group showed higher scores on HAMD (*F*_2,107_ = 25.366, *p* < 0.001) and YMRS (*F*_2,107_ = 13.206, *p* < 0.001) than both AO and HC groups (all *p*_corrected_ < 0.001 after Bonferroni correction), while the latter two groups did not differ from each other (*p*_corrected_ = 1, Table 1, Figure 1A). Similar between‐group differences were observed on C3 level (*F*_2,108_ = 23.651, *p* < 0.001), such that the SO group showed significantly lower C3 level than both AO and HC groups (all *p*_corrected_ < 0.001 after Bonferroni correction), while no significant difference was observed between the AO and HC groups (*p*_corrected_ = 0.063, Table 1, Figure 1B).

**TABLE 1:** Demographic, clinical variables, and C3 levels of participants.

	SO, *N* = 31	AO, *N* = 39	HC, *N* = 41	Statistics
**Mean (SD)/*n* (%)**				
Age, years	18.31 (5.05)	18.54 (4.64)	16.56 (3.24)	*F*_2,108_ = 2.480, *p* = 0.088^a^
Sex, female %	17 (54.8%)	22 (56.4%)	21 (51.2%)	*χ* ^2^ = 0.228, *p* = 0.892^b^
Education, years	10.00 (3.82)	10.90 (3.25)	10.07 (2.81)	*F*_2,108_ = 0.870, *p* = 0.422^c^
HAMD	7.93 (9.33)	0.46 (0.91)	0.39 (0.97)	*F*_2,107_ = 25.366, *p* < 0.001^a^
				SO > AO: *p*_corr_ < 0.001^d^ SO > HC: *p*_corr_ < 0.001^d^ AO > HC: *p*_corr_ = 1^d^
YMRS	2.03 (2.80)	0.36 (1.37)	0.02 (0.16)	*F*_2,107_ = 13.206, *p* < 0.001^a^
				SO > AO: *p*_corr_ = 0.003^d^ SO > HC: *p*_corr_ = 0.009^d^ AO > HC: *p*_corr_ = 1^d^
C3 (Ln transformed)	12.13 (0.66)	12.84 (0.81)	13.26 (0.47)	*F*_2,108_ = 23.651, *p* < 0.001^a^
				SO < AO: *p*_corr_ < 0.001^d^ SO < HC: *p*_corr_ < 0.001^d^ AO < HC: *p*_corr_ > 0.05^d^

*Note:* Group difference in clinical variables and C3 levels, adjusted for age and sex.

Abbreviations: AO, asymptomatic offspring; C3, complement component 3; HAMD, the Hamilton Depression Rating Scale; HC, healthy controls; SO, symptomatic offspring; YMRS, the Young Mania Rating Scale.

^a^ Nonparametric Kruskal–Wallis test.

^b^ Chi‐square test.

^c^ One‐way ANOVA test.

^d^ Post hoc pairwise comparisons with Bonferroni correction.

### 3.2 Seed‐Based FC Between‐Group Conjunction Analysis

Based on the general linear model which compared the hippocampus FC across the SO, AO, and HC groups, we first revealed that the SO group showed significantly reduced FC compared to the HC group, between the left hippocampus and a cluster in the left cerebellum (peak MNI coordinate = −42, −78, −27, voxel size = 19, TFCE = 463.7, *p*_FWE_ = 0.039, Figure 1C). No significant difference was observed for the right hippocampus FC between SO and HC groups (*p*_FWE_ > 0.1). Following the conjunction analysis procedure (see Section 2), we then evaluated the difference between SO and AO groups within an 8‐mm sphere centered on the peak coordinate of the above left cerebellum cluster. This analysis revealed a significant left cerebellar region where hippocampus FC was also reduced in the SO group compared to the AO group (peak MNI coordinate = −42, −78, −21, voxel size = 51, TFCE = 98.45, *p*_FWE_ = 0.001). Follow‐up analyses on the mean left hippocampus FC extracted from this region revealed that the SO group showed lower FC than both AO (*t* = −3.67, *p* < 0.001, *p*_corrected_ = 0.002 after Bonferroni correction) and HC groups (*t* = −3.79, *p* < 0.001, *p*_corrected_ = 0.001), after controlling for age and sex (Figure 1D).

### 3.3 DCM Analysis

**FIGURE 2:**
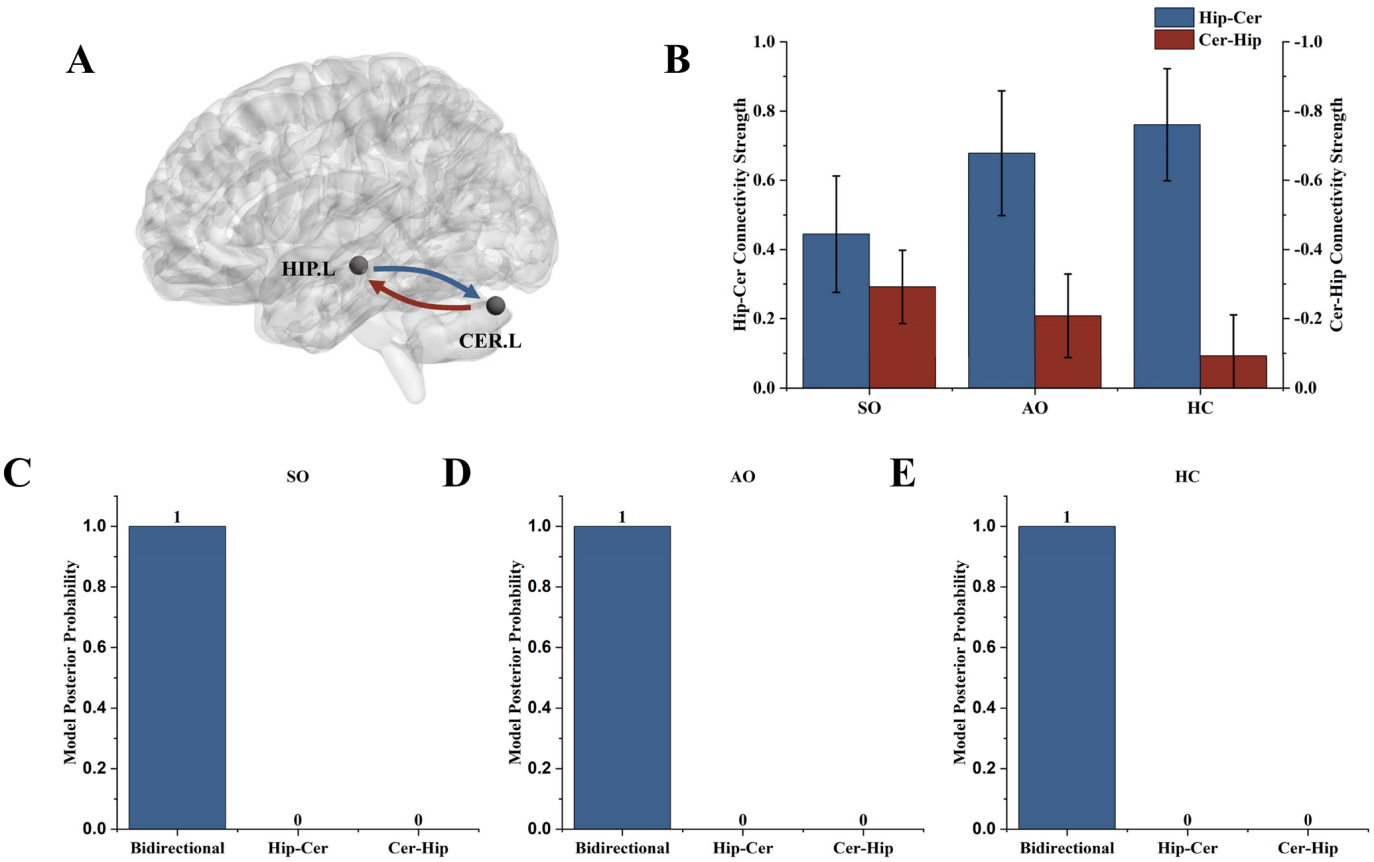
Directional effective connectivity between the left hippocampus and the left cerebellum. (A) Regions of interest overlain on a glass brain. (B) The resting‐state connectivity directed from the left hippocampus to the left cerebellum is significantly positive in all three groups (SO, AO, and HC), while the connectivity directed from the left cerebellum to the left hippocampus is significantly negative in the SO group, marginally negative in the AO group, and nonsignificant in the HC group. (C–E) Bayesian model selection procedure revealed the best model in each group was the one denoting bidirectional connectivity between the left hippocampus and the left cerebellum. The numbers above bars represent model posterior probability. Models with probability ≥ 90% are typically considered as receiving significant support. Both mean and ±1 standard error of the mean are presented. AO, asymptomatic offspring; CER.L, left cerebellum; HC, healthy control; HIP.L, left hippocampus; SO, symptomatic offspring.

According to the initial model diagnostic, the DCM models explained reasonable variance of the seed time series of all (> 20%) but five participants (2 AOs, 2 SOs and 1 NC). Also, all participants showed largest connectivity well above the baseline of 1/8 hz, indicating that model fitting was satisfactory. Excluding the five participants, the best model for all groups was the one denoting bidirectional connectivity between the left hippocampus and the left cerebellum (Figure 2A). The bidirectional model received 100% evidence (posterior probability) in all the SO, AO, and HC groups (Figure 2C–E). In all groups, the hippocampus‐to‐cerebellum connectivity is significantly positive (SO: *t*_28_ = 2.644, *p* = 0.013; AO: *t*_36_ = 3.769, *p* = 0.001; HC: *t*_39_ = 4.692, *p* < 0.001), while the cerebellum‐to‐hippocampus connectivity is significantly or marginally negative in the SO (*t*_28_ = −2.753, *p* = 0.010) and AO groups (*t*_36_ = −1.723, *p* = 0.093), and nonsignificant in the HC group (*t*_39_ = −0.782, *p* = 0.439) (Figure 2B). After controlling for age and sex, no between‐group difference was observed for the hippocampus‐to‐cerebellum connectivity (*F*_2,101_ = 0.873, *p* = 0.42), or for the cerebellum‐to‐hippocampus connectivity (*F*_2,101_ = 0.548, *p* = 0.58).

### 3.4 Correlation Analysis Between Clinical Variables, C3 and Hippocampus FC

#### 3.4.1 C3 & FC

Across all participants, higher C3 levels were associated with more positive FC between the left hippocampus seed and the left cerebellum region obtained from the conjunction analysis (Spearman *rho* = 0.285, *p* = 0.002), after controlling for age and sex (Figure 1E).

#### 3.4.2 C3 & Clinical Variables

Across all participants, higher C3 levels were significantly associated with lower HAMD scores (Spearman *rho* = −0.343, *p* < 0.001) (Figure 1F), after controlling for age and sex. No significant correlation was observed between C3 levels and YMRS scores (*p* > 0.2).

#### 3.4.3 FC & Clinical Variables

Across all participants, more positive left hippocampus FC with the left cerebellum region was associated with lower HAMD (Spearman *rho* = −0.222, *p* = 0.020) (Figure 1G) and YMRS scores (Spearman *rho* = −0.206, *p* = 0.031), after controlling for age and sex.

All the above significant correlations survived multiple‐testing correction using the Holm‐Bonferroni method (all *p*_corrected_ < 0.05).

### 3.5 Mediation and Moderated Mediation Analyses

**FIGURE 3:**
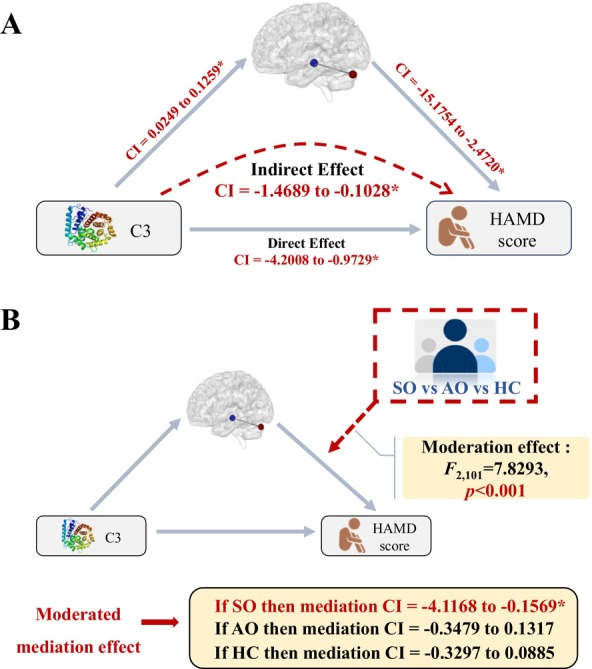
Mediation and moderated mediation models of the C3‐hippocampal FC‐HAMD score relationship. (A) In the combined samples, the left hippocampus‐left cerebellum FC significantly and partially mediated the negative association between C3 level and HAMD score. Specifically, the C3 level exhibited a positive correlation with the left hippocampus‐left cerebellum FC, which in turn showed a negative correlation with the HAMD score. (B) The group variable further significantly moderated the mediation model. Specifically, the mediation effect of the left hippocampus‐left cerebellum FC on the negative relationship between the C3 level and the HAMD score was only observed in the SO group, but not in the AO or HC group. CI, Bootstrapping confidence interval. *Significant at *p* < 0.05.

Since we observed significant pairwise correlations between the C3 level, the left hippocampus‐left cerebellum FC, and HAMD scores across all participants, we proceeded to examine whether the left hippocampus‐left cerebellum FC mediated the significant association between C3 level and HAMD scores. This analysis revealed a significant negative partial mediation effect (bootstrapping 95% CI = −1.4689 to −0.1028, Figure 3A). Specifically, C3 level was positively associated with left hippocampus‐left cerebellum FC (bootstrapping 95% CI = 0.0249 to 0.1259), which in turn was negatively associated with HAMD scores (bootstrapping 95% CI = −15.1754 to −2.4720) (Figure 3A).

Furthermore, we examined whether this mediation effect was different across the SO, AO, and HC groups, through conducting a moderated mediation analysis, in which the group variable acted as the moderator coded as a multi‐categorical variable. This analysis revealed a significant moderating effect of group (*F*_2,101_ = 7.8293, *p* < 0.001). Specifically, the left hippocampus‐left cerebellum FC significantly mediated the negative association between C3 level and HAMD score only in the SO group (bootstrapping 95% CI = −4.1168 to −0.1569), but not in the AO (bootstrapping 95% CI = −0.3479 to 0.1317) or HC group (bootstrapping 95% CI = −0.3297 to 0.0885) (Figure 3B).

## 4 Discussion

This study provides insights into the neuroinflammatory pathway underpinning the heightened risk of BD offspring who also exhibit subthreshold mood symptoms. To our knowledge, this is the first evidence indicating a critical mediating role of hippocampal functional connectivity for the relationship between complement C3 levels and mood symptoms specifically among individuals with both familial and symptomatic risks. Thus, our findings demonstrate distinct C3‐brain‐mood symptom pathways among individuals with differential risks for BD and other mood disorders. As such, this new evidence contributes to the recent accumulating literature on the neuroinflammatory processes associated with the intricate synergistic effect of familial and symptomatic risks of mood disorders [6, 7].

Multiple previous studies demonstrated that inflammatory processes in the brain play pivotal roles in the development of BD [5]. Inflammatory processes are closely regulated by the body's complement protein system, within which C3 is a central component that is intensively involved in the regulation of pro‐inflammatory responses [11]. Previous investigations have consistently reported C3 abnormalities in BD patients [40, 41]. However, there has been limited evidence on C3 abnormality in high‐risk BD offspring. Our results revealed that individuals with both familial and symptomatic risks exhibited significantly lower C3 levels compared to both individuals with only familial risk and to HC. This is consistent with recent evidence implicating reduced C3 levels in high‐risk psychotic individuals [42]. These findings collectively show that reduced C3 levels may occur prior to the first onset of psychiatric disorders, particularly among individuals already exhibiting subthreshold symptoms.

It had been previously shown that C3 level alterations are associated with mood symptoms and abnormal synaptic pruning [15, 16, 43]. For instance, abnormal fear and anxiety behaviors were observed in C3‐deficient mice, who also exhibited social interaction deficits and repetitive behaviors [16, 44]. In addition, the reduced expression of C3 in the mouse cerebellum was associated with decreased synaptic density [43], which had been found to be inversely associated with the severity of depressive symptoms [45]. Moreover, reduced serum C3 levels may lead to synaptic pruning dysregulation and were linked with severe depression and anxiety symptoms in systemic lupus erythematosus patients [15]. Our current findings extended the existing research by showing that peripheral C3 levels are inversely associated with depressive symptoms among young individuals with differential risks for BD, thereby providing further evidence supporting a critical role of C3 in regulating the negative emotion system.

The observed lower FC between the left hippocampus and left cerebellum among individuals with both BD familial and symptomatic risks, compared to those with only familial risk and HC, indicates a distinct neurobiological profile specifically associated with the synergistic effect of multiple BD risks. The hippocampus is a central component of the limbic system and constitutes part of the brain's default mode network [46]. Through extensive anatomical and functional connections with other brain regions closely involved in emotion regulation such as the prefrontal cortex, cerebellum, and striatal regions, the hippocampus is conveniently situated to be a central structure in the regulation of negative emotions [47]. Previous evidence indicated reduced resting‐state functional connectivity between the hippocampus and the cerebellum in symptomatic BD offspring, which might underpin their emotion dysregulation [20]. The cerebellum functional connectivity alteration that we observed in symptomatic BD offspring was mainly localized in the posterior cerebellar lobe (Crus I), which had been consistently linked to cognitive control and emotion regulation functions [48]. Furthermore, our DCM analysis revealed that across the symptomatic, non‐symptomatic offspring, and HC, the functional connectivity between the left hippocampus and left cerebellum was bidirectional. Specifically, the connectivity directed from the hippocampus to the cerebellum is excitatory, while the reverse connectivity was inhibitory, and may underlie a fundamental regulatory mechanism in BD offspring. The hippocampal excitatory influence on the cerebellum could be integral to the cognitive processes underlying emotional regulation, potentially enhancing its ability to contribute to the regulation of emotional responses [49]. Conversely, the inhibitory cerebellar feedback to the hippocampus might modulate emotional responses, contributing to the stability of affective states [50] and improving cognitive functions such as memory recall and spatial mapping [49, 51]. These findings suggest that the intrinsic communication pattern between the hippocampus and the cerebellum is relatively stable and invariant to BD risks.

Past evidence indicates that the functional connectivity between the hippocampus and the cerebellum was higher in BD patients in remission and their unaffected relatives, but decreased in individuals experiencing manic or depressive episodes [52, 53, 54]. These findings indicate that while dysconnectivity between the hippocampus and the cerebellum may underpin the emotion regulation deficits of patients with BD or Major Depression, elevated connectivity between these regions could signal compensatory or resilience mechanisms that allow remitted patients and unaffected relatives to stay well. Our findings further demonstrated that only BD offspring who simultaneously exhibited subthreshold mood symptoms showed a decrease in hippocampus‐cerebellum functional connectivity, which could underpin these individuals' particularly heightened vulnerability to subsequently developing mood disorders [20]. On the contrary, the hippocampus‐cerebellum functional connectivity strength was comparable in asymptomatic BD offspring and HC, suggesting a normative level of functional interaction between these brain structures that represents a resilience mechanism against familial risk.

Consistent with our hypotheses, we found that the hippocampus‐cerebellum functional connectivity partially mediated the negative relationship between C3 level and depressive symptoms. However, this mediation effect was exclusively observed in symptomatic BD offspring, but not in asymptomatic BD offspring or HC. While the precise pathway via which C3 influences the hippocampal functional connectivity remains unclear, recent evidence indicates that the activation of C3 can affect the structure and functionality of the hippocampus [22], which are in turn implicated in the development of various psychiatric disorders such as schizophrenia and autism [16]. Furthermore, increased numbers of hippocampal synapses had been observed in C3‐deficient mice and were associated with abnormalities in hippocampal‐dependent learning [55].

It was unclear why the hippocampus‐cerebellum functional connectivity mediated the negative effect of C3 level on depressive symptoms only among symptomatic BD offspring. Several potential reasons might account for this finding. First, since our AO sample showed no observable abnormality in C3 level, hippocampus‐cerebellum connectivity, or depressive symptoms compared to HC, there might not be sufficient symptomatic or neuroinflammatory fluctuations within these samples to detect significant mediation effects. Second, depressive symptom is a core clinical manifestation of BD offspring and a major predictive factor for future conversion to full‐blown mood disorder. Given both the hippocampus and its functional interaction with the cerebellum play major roles in the regulation of negative emotions [47, 56], it is likely that the hippocampus‐cerebellum connectivity is particularly important in determining the level of depressive symptoms in symptomatic individuals. Third, the effect of the C3 system on hippocampal function and structure could be modulated by other pertinent factors such as stress, which has been repeatedly found to be a major predisposing factor for mood disorders including BD [57]. Chronic stress activates the hypothalamus‐pituitary‐adrenal (HPA) axis, leading to the release of glucocorticoids which in turn can disrupt the balance of C3 and synaptic connections in the hippocampus [22, 58]. Thus, stress‐induced synaptic changes in the hippocampus may interact with the complement C3 system in determining hippocampal functions in emotion regulation [59], and higher levels of chronic stress among SO might contribute to the observed mediation effect.

## 5 Limitation

Several limitations of the current study need to be considered. First, the cross‐sectional design of the current study precludes ascertaining the directionality of relationships and causal inferences. The longitudinal part of the current project is ongoing, which will allow us to elucidate the temporal dynamic relationships between C3, brain connectivity, and symptom changes. Second, we focused on the functional connectivity of the hippocampus. Although such focus was informed by previous research on BD risk, future research should extend our approach to other brain structures implicated in emotion regulation. Third, future study could incorporate measurements of major inflammatory cytokines such as CRP and IL6 in order to delineate the relationship between C3, inflammation, and brain function in more detail. Future research could also include more direct measures of neuroinflammation, such as cerebrospinal fluid analysis.

## 6 Conclusion

This study provides novel evidence for a specific C3‐brain‐mood symptom pathway among individuals with both familial and symptomatic risks for mood disorders. This pathway highlights a critical role of the hippocampus‐cerebellum functional connectivity as the intermediate brain mechanism bridging the relationship between C3 and depressive symptoms. Our findings add to the recent growing literature on the neuroinflammatory pathways underpinning high‐risk for major psychiatric illnesses, and indicate new biological targets for early detection, prevention, and intervention of particularly vulnerable individuals with a high chance to develop mood disorders.

## Author Contributions

K.L. conceptualized the project. W.Z., X.L., W.L., S.W., and K.L. implemented the project. S.W., Z.L., and R.S. managed the data and performed the data analysis. S.W., Z.L., R.S., and K.L. drafted the manuscript. S.W. and Z.L. constructed the figures. J.C., S.Y., and K.L. provided critical comments on the final version of the manuscript.

## Conflicts of Interest

The authors declare no conflicts of interest.

## Supporting information


**Figure S1:** bdi70056‐sup‐0001‐FigureS1.pdf.

**Data S1:** bdi70056‐sup‐0002‐Supinfo1.docx.

## Data Availability

Data will be made available on request from the corresponding author (K.L., klin@gzhmu.edu.cn).
