# Supplementary figures and images for: The Role of the Complement C3‐Hippocampus Pathway in Relation With Mood Symptoms in Offspring of Parents With Bipolar Disorder
Source: Bipolar Disord. 2025 Sep 1;27(6):461–71. doi: 10.1111/bdi.70056 (PMC12483309; doi:10.1111/bdi.70056)

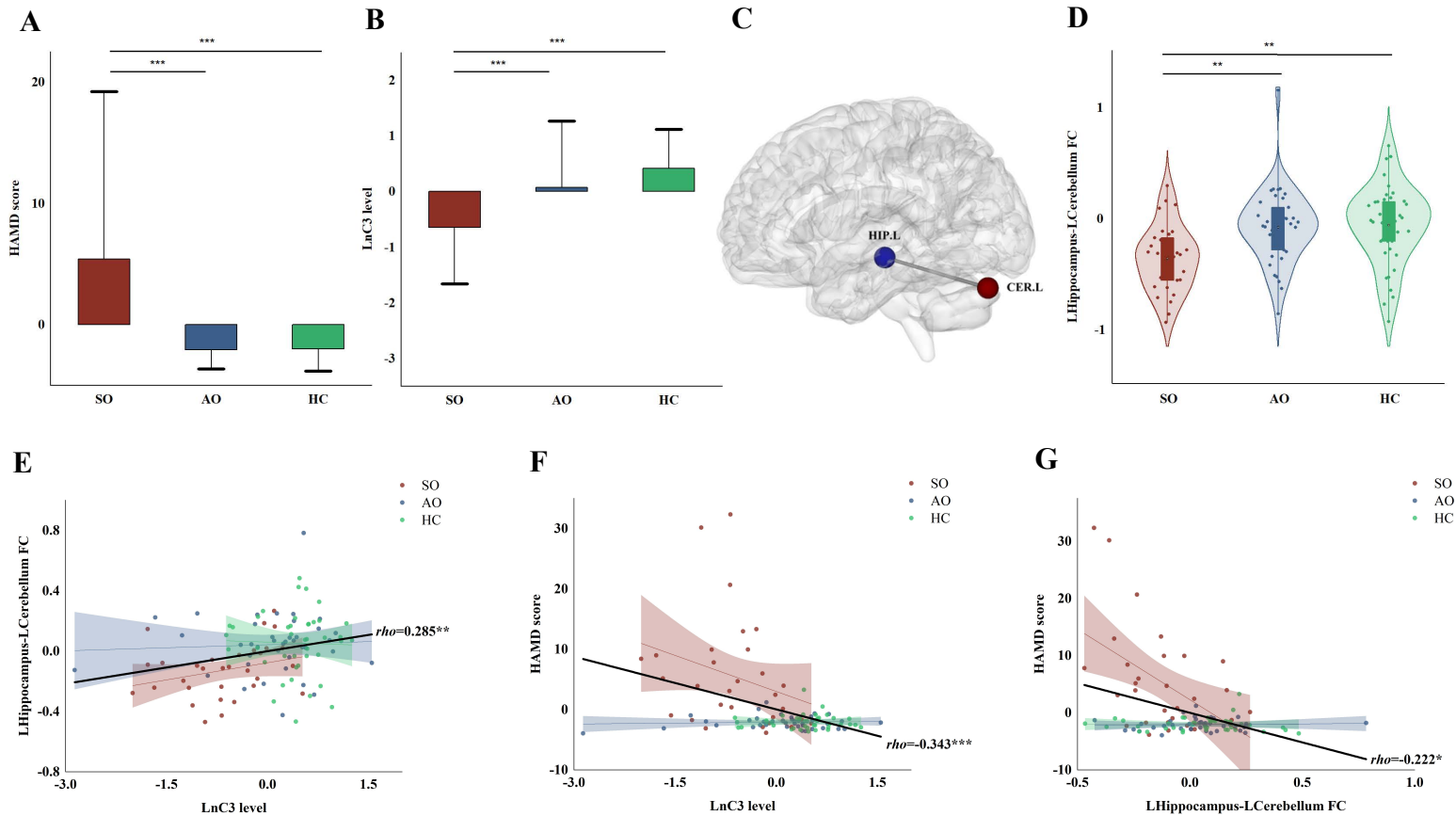

Supplement: Supplementary file 1 — Figure S1: bdi70056‐sup‐0001‐FigureS1.pdf. [file BDI-27-461-s001.pdf]
