# Supplementary material for: The Role of the Complement C3‐Hippocampus Pathway in Relation With Mood Symptoms in Offspring of Parents With Bipolar Disorder
Source: Bipolar Disord. 2025 Sep 1;27(6):461–71. doi: 10.1111/bdi.70056 (PMC12483309; doi:10.1111/bdi.70056)
Supplement: Supplementary file 2 — Data S1: bdi70056‐sup‐0002‐Supinfo1.docx. [file BDI-27-461-s002.docx]

**Supplementary Material**

**Participants assessment**

The present study was derived from the Recognition and Early intervention on Prodromal Bipolar Disorder (REI-PBD) project, which was founded by the Global Mood and Brain Science Initiative in 2013. This study was reviewed and approved by the Institutional Review Board of the Affiliated Brain Hospital of Guangzhou Medical University. All participants and/or their guardians provided written informed consent.

BD offspring had parents who were diagnosed with bipolar type I or type II disorder according to the Diagnostic and Statistical Manual of Mental Disorders (DSM)-IV criteria. The symptomatic offspring was defined as observation of at least 1 of the following subthreshold syndromes: 1) exhibiting two or three hypomanic symptoms for at least 4 days (not meeting the DSM-IV hypomania episode criteria); 2) presenting two or more symptoms of a major depressive episode for at least 1 week but not meeting the DSM-IV major depressive episode criteria (falling short of the required number of symptoms or duration of 2 weeks); and 3) one or more attenuated psychotic symptoms present for at least 10 minutes for each manifestation, occurring 2-7 times per week for at least 3 months. The attenuated psychotic symptoms included: odd ideas and beliefs, ideas of reference, bizarre thoughts or speech, unusual perceptual experiences, grandiosity, suspicious ideas, paranoid ideas, odd mannerisms, hallucinations, disorganized/catatonic behaviors; 4) two or more hyperactivity/impulsivity symptoms of attention deficit hyperactivity disorder (ADHD) as observable by teachers, peers, and/or parents. All symptomatic offspring who manifested ADHD or attenuated psychotic symptoms also needed to displayed either sub-threshold depressive or hypomanic symptoms.

All participants received systematic interviews and clinical scales to assess their symptom levels, including the Kiddie Schedule for Affective Disorders and Schizophrenia for School Aged Children-Parent Lifetime Version (K-SADS-PL, for participants under 18 years old), the Structured Clinical Interview for DSM-IV Axis II Disorders (SCID-II, for adult participants), the Hamilton Anxiety Rating Scale (HAMA), the Hamilton Depression Rating Scale (HAMD), the Young Mania Rating Scale (YMRS), the Brief Psychiatric Rating Scale (BPRS), and the Global Assessment of Functioning Scale/Children’s Global Assessment of Functioning Scale (GAS/CGAS).

**Resting-State data head motion control**

To control for head motion, we first excluded participants with maximum head displacement > 2.0mm or rotation degree > 2°. Fourteen participants (6 SO, 8 AO) were excluded due to excessive motion. Second, the data were scrubbed based on the FD value at the default threshold of 0.5. Third, the Friston-24 motion parameters were generated during realignment and, along with the scrubbing regressors, were regressed out from the data to further minimize the motion effect. Finally, Kruskal-Wallis test showed that the AO, SO, and HC groups showed no difference on the mean FD value (*p*=0.83) or the number of scans scrubbed (*p*=0.47). Thus, any between-group differences were unlikely to be due to motion effects.
